# A Pilot Study of ^18^F‐rhPSMA‐7.3‐PET/MRI to Reduce Mischaracterization of Active Surveillance and Focal Therapy Candidates With Occult Higher Risk Disease

**DOI:** 10.1002/pros.70147

**Published:** 2026-03-15

**Authors:** Ridwan Alam, Derek Hesse, Nikki Hubbard, Emma McGarrity, Sai Kumar, Clayton Neill, Yutai Li, Nicole Handa, Hiten D. Patel, Edward M. Schaeffer, Hatice Savas, Ashley E. Ross

**Affiliations:** ^1^ Department of Urology Northwestern University Feinberg School of Medicine Chicago Illinois USA; ^2^ Department of Radiology Northwestern University Feinberg School of Medicine Chicago Illinois USA

**Keywords:** active surveillance, focal therapy, prostate cancer, PSMA‐PET, rhPSMA

## Abstract

**Introduction:**

PSMA‐PET offers an opportunity to reduce the mischaracterization of disease in active surveillance (AS) and focal therapy (FT) candidates. We describe the results of a pilot clinical trial evaluating ^18^F‐radiohybrid(rh)PSMA‐7.3‐PET/MRI to detect occult adverse pathology among potential AS and FT candidates (NCT05852041).

**Methods:**

We enrolled 20 men with low risk or favorable intermediate risk prostate cancer and Decipher score ≥ 0.45 diagnosed after an MRI‐informed prostate biopsy. All patients underwent PSMA‐PET/MRI followed by either PET/MRI‐guided biopsy or radical prostatectomy within 90 days. The outcome of interest was detection of grade group (GG) 3–5 disease, seminal vesicle invasion (pT3b), or lymph node involvement (pN1). A detection rate of 15% for this outcome was considered clinically significant. Management decisions and confidence in those decisions were recorded before and after the scan using a 3‐point scale. A paired *t*‐test was performed to compare the change in confidence decisions.

**Results:**

At enrollment, 17 patients (85%) had favorable intermediate risk and 3 (15%) had low risk prostate cancer. The median Decipher score was 0.58 (IQR 0.49–0.65). Five patients (25%) demonstrated the outcome of interest based on upgrading alone. None had upstaging to pT3b or pN1. Major changes in management plan occurred in 7 patients (35%). Average confidence in decisions improved from moderate (2.05) to high (2.80) after the scan (*p* < 0.001).

**Conclusions:**

^18^F‐rhPSMA‐7.3‐PET/MRI can detect occult higher risk disease in men who are otherwise candidates for AS or FT. The scan prompted major changes in management and increased confidence in the final treatment strategy.

## 1 Introduction

Outcomes for localized prostate cancer vary greatly and can be prognosticated based on disease features at presentation. As such, risk stratification has become a fundamental component in the management of prostate cancer, with higher risk presentations commanding increasingly intensive therapies [1, 2]. This allows for a more tailored approach that better balances the harms and benefits of treatment at each point along the disease spectrum. In particular, the utilization of active surveillance (AS) and focal therapy (FT) for patients with favorable risk disease has played an integral role in combating the overtreatment of prostate cancer.

AS protocols are intentionally designed to maintain a large window of curability. Indeed, the rates of metastasis and prostate cancer‐specific mortality for patients on AS are exceedingly low [3]. Still, there is a desire to avoid early reclassification to grade group (GG) ≥ 3, which has been coined “extreme reclassification” and typically implies the need for intensified treatment. Even in AS populations comprised primarily of GG 1 disease, extreme reclassification represents about one‐third of all reclassification events [4]. This proportion can be expected to rise as AS is increasingly employed in patients with GG 2 disease [5]. Currently, approximately 25%–40% of patients progress to treatment within a few years of initiating AS [3, 4, 6]. This suggests that there is still considerable room to optimize the selection process for potential AS candidates. Many of the same concerns that plague selection for AS also affect FT, particularly with respect to the presence of disease not detected on imaging or biopsy [7, 8].

Advanced tools have been developed to assist with patient selection. The Decipher Prostate genomic classifier, for example, can identify men at higher risk of disease progression [9, 10, 11]. Multiparametric magnetic resonance imaging (mpMRI) additionally aids in the understanding of disease burden, but false negatives and misclassifications of disease risk still occur [12, 13]. Along these lines, there is growing data to support that prostate specific membrane antigen‐positron emission tomography (PSMA‐PET)/MRI may provide greater spatial resolution and anatomic detail of the prostate over conventional mpMRI alone [14, 15]. Despite this, PSMA‐PET is largely reserved for the detection of metastatic disease and not for the evaluation of localized disease.

We propose that PSMA‐PET/MRI offers the potential to detect higher risk disease and reduce early reclassification or recurrence among seemingly suitable AS and FT candidates. Here, we describe the interim results of a pilot clinical trial evaluating the utility and safety of ^18^F‐radiohybrid(rh)PSMA‐7.3‐PET/MRI to detect occult adverse pathology among potential AS and FT candidates.

## 2 Methods

This is a single institution pilot clinical trial with a goal to accrue 20 patients (NCT05852041). All patients provided written informed consent prior to enrollment. This study was approved by the Northwestern University Institutional Review Board (STU00218970).

**Figure 1:**
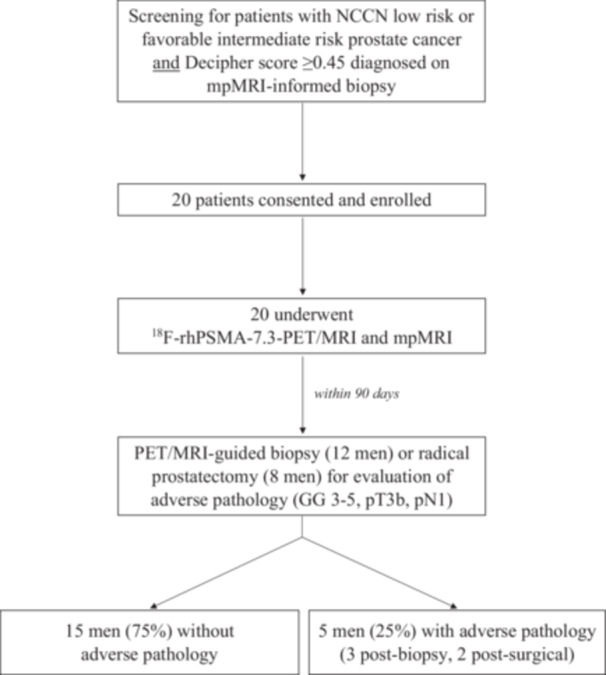
Consolidated Standards of Reporting Trials (CONSORT) flow diagram of study schema and primary endpoint.

From October 2023 to September 2024, we screened adult men aged ≥ 18 years with low risk or favorable intermediate risk prostate cancer, as defined by the National Comprehensive Cancer Network (NCCN), and Decipher score ≥ 0.45 diagnosed at the time of an mpMRI‐informed biopsy (Figure 1). A minimum 10‐core biopsy was required to be considered for enrollment. Patients with a history of any therapy for prostate cancer or surgical therapy for benign prostatic hyperplasia were excluded. Use of any 5α‐reductase inhibitors in the 30 days prior to screening was prohibited. Men with a history of hip replacement or other pelvic hardware which may obscure visualization of the prostate were also excluded.

Baseline clinicopathologic characteristics were recorded for each patient. All patients were imaged using both ^18^F‐rhPSMA‐7.3‐PET/MRI and mpMRI in the same session. A 300 megabecquerel (MBq) dose of ^18^F‐rhPSMA‐7.3 (Blue Earth Diagnostics, Inc., Needham, MA) was administered as an intravenous bolus 60 min ± 10 min prior to the scan. Gadolinium‐based contrast agents were administered per routine protocol for the mpMRI. All PET/MRI images were interpreted by a double board‐certified chest/abdominal imaging and nuclear medicine trained physician (H.S.) with extensive experience in prostate imaging. Images were interpreted using Visage and syngo.via Picture Archiving and Communication System (PACS) for better image fusion of simultaneous PET and MRI sequences. The Philips DynaCAD system was used for lesion annotation, and the Philips UroNav system was used for the fusion biopsy using a transperineal approach. Adverse events (AE) occurring within 24 h of injection of ^18^F‐rhPSMA‐7.3 were recorded in accordance with Common Terminology Criteria for Adverse Events (CTCAE) version 5.0.

All patients subsequently underwent either PET/MRI‐guided biopsy or radical prostatectomy within 90 days of imaging. The primary outcome of interest was the detection of GG 3–5 disease, seminal vesicle invasion (pT3b), or lymph node involvement (pN1) on post‐imaging pathology. A detection rate of 15% (i.e., 3 patients) for this outcome was considered clinically significant.

To evaluate the pragmatic impact of PET/MRI on clinical decision making, we reviewed management decisions before and after the scan. Changes in management were denoted as none, minor (e.g., FT to whole gland therapy), or major (e.g., surveillance to treatment). Confidence in each decision was recorded using a 3‐point scale (1 = low, 2 = moderate, and 3 = high). A paired *t*‐test was performed to compare the change in confidence.

All statistical analyses were performed using STATA v18.0 (StataCorp, College Station, TX) with statistical significance set at *α* = 0.05.

## 3 Results

We enrolled 20 patients in this study with a median age of 61.9 years (IQR 56.8–68.7) and median PSA of 4.8 ng/mL (IQR 4.3–6.5) (Table 1). Our cohort was comprised of 17 White patients (85%) and 3 Black patients (15%). Using the NCCN criteria, there were 17 patients (85%) with favorable intermediate risk disease and 3 patients (15%) with low risk disease. The median Decipher score was 0.58 (IQR 0.49–0.65). Nine patients (45%) had high risk (> 0.60) and 11 patients (55%) had intermediate risk (0.45–0.60) Decipher scores.

**Table 1:** Baseline clinicopathologic characteristics of patients enrolled in the study.

Baseline characteristics	Study cohort (*n* = 20)
Age, years (IQR)	61.9 (56.8–68.7)
Race, *n* (%)	
White	17 (85%)
Black	3 (15%)
Charlson Comorbidity Index, *n* (%)	
1–3	4 (20%)
4–6	10 (50%)
7–9	6 (30%)
PSA, ng/mL (IQR)	4.8 (4.3–6.5)
Prostate volume, g (IQR)	39.5 (28.8–55.8)
PSA density, ng/mL/g (IQR)	0.12 (0.08–0.19)
Clinical stage, *n* (%)	
T1c	19 (95%)
T2a	1 (5%)
PI‐RADS score, *n* (%)	
1–2	2 (10%)
3	3 (15%)
4	12 (60%)
5	3 (15%)
Grade group, *n* (%)	
1	4 (20%)
2	16 (80%)
NCCN risk category, *n* (%)	
Low	3 (15%)
Favorable intermediate	17 (85%)
Decipher score, points (IQR)	0.58 (0.49–0.65)
Decipher category, *n* (%)	
Intermediate (0.45–0.60)	11 (55%)
High (> 0.60)	9 (45%)

### 3.1 Detection of Adverse Pathology

**Figure 2:**
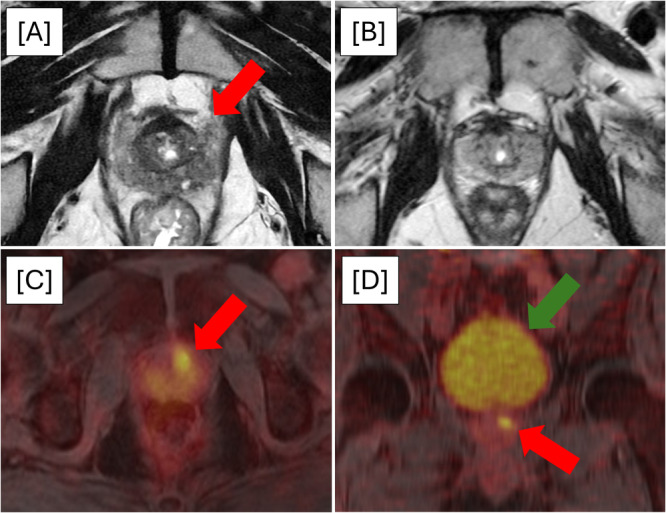
Representative images of a patient enrolled in the study. (A) Pre‐enrollment mpMRI shows a PI‐RADS 3 lesion in the left anterior midgland peripheral zone (red arrow), and subsequent MRI‐guided biopsy revealed GG 2 disease. (B) Study mpMRI shows no visible lesions. (C) Axial image of ^18^F‐rhPSMA‐7.3‐PET/MRI demonstrates a PET‐avid lesion in the left anterior midgland peripheral zone (red arrow). PET/MRI‐guided biopsy revealed GG 4 disease. (D) Coronal image of ^18^F‐rhPSMA‐7.3‐PET/MRI demonstrating the minimal scatter artifact from urinary excretion (green arrow) and the distinctly visible PET‐avid lesion in the prostate (red arrow). [Color figure can be viewed at wileyonlinelibrary.com]

The median time from pre‐enrollment mpMRI to PSMA‐PET/MRI was 4.7 months (IQR 4.1–6.2). Nineteen patients (95%) demonstrated PET‐avid lesions within the prostate (Figure 2). The post‐imaging pathologic specimens were obtained from a PET/MRI‐guided biopsy in 12 patients (60%) and from a radical prostatectomy in 8 patients (40%). The median time from pre‐enrollment biopsy to post‐imaging pathology was 5.1 months (IQR 4.0–6.2).

Five patients (25%) demonstrated the outcome of interest based on upgrading alone: GG 1 to GG 3 (*n* = 1), GG 1 to GG 5 (*n* = 1), GG 2 to GG 3 (*n* = 2), and GG 2 to GG 4 (*n* = 1). PET‐avid lesions were present in all 5 men with these outcomes. Three of these outcomes were obtained on biopsy with the remaining 2 from radical prostatectomy. Of the 8 patients who underwent surgery, none had upstaging to pT3b or pN1. The singular patient who did not demonstrate an intraprostatic PET‐avid lesion underwent radical prostatectomy and did not meet the outcome of interest (pT2N0 GG 2).

PET‐positive lesions not detected on pre‐enrollment mpMRI occurred in 5 patients (25%). In 2 of these patients, the mpMRI was considered negative (PI‐RADS 1 or 2). The other 3 patients had PET‐avid lesions located in areas that were distinct from the PI‐RADS 3–5 lesions. Three of the patients were upgraded (GG 1 to GG 2, GG 1 to GG 3, and GG 2 to GG 3), each of which was associated with a change in NCCN risk classification. The remaining 2 patients were found to have GG 2 disease at both pre‐enrollment biopsy and post‐imaging pathology.

### 3.2 Decision Making

**Figure 3:**
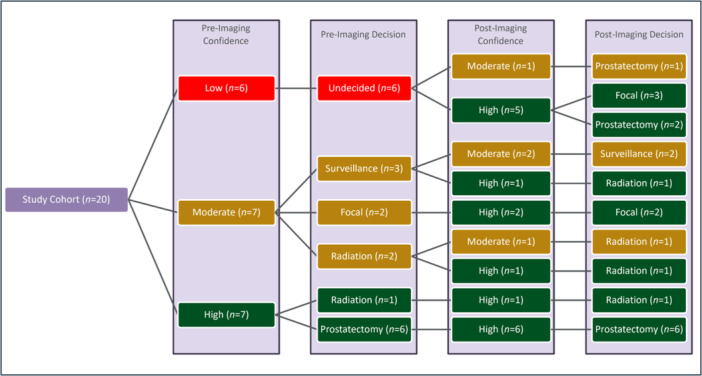
Flow diagram of confidence associated with management decisions before and after imaging with ^18^F‐rhPSMA‐7.3‐PET/MRI. [Color figure can be viewed at wileyonlinelibrary.com]

Prior to PSMA‐PET/MRI, clinicians and patients were undecided with low confidence regarding the initial management strategy in six cases (30%) (Figure 3). In the remaining 14 cases, joint decision making demonstrated moderate or high confidence in strategies, including AS (*n* = 3), FT (*n* = 2), radical prostatectomy (*n* = 6), and radiation (*n* = 3).

After the scan, major changes in management occurred in 7 patients (35%). Six of these patients were undecided prior to the scan and moved toward definitive therapy, with radical prostatectomy and FT each chosen by 3 patients. The seventh patient moved from AS with moderate confidence to radiation therapy with high confidence.

Of the 5 patients with PET‐positive lesions not detected on pre‐enrollment mpMRI, a major change in management occurred in 3. The changes were as follows: undecided to FT, undecided to radical prostatectomy, and AS to radiation.

None of the 20 patients demonstrated low confidence in the final treatment plan after undergoing PSMA‐PET/MRI. All 5 patients who ultimately chose FT did so with a high degree of confidence. Overall, the average confidence in decisions improved from moderate (2.05) to high (2.80) as a result of the scan (*p* < 0.001).

### 3.3 Safety

Four patients (20%) experienced Grade 1 AEs that were considered possibly related to the administration of ^18^F‐rhPSMA‐7.3. Three patients reported nausea, and 1 patient reported both nausea and headache. All events resolved spontaneously with no sequelae within 24 h.

## 4 Discussion

The use of PSMA‐PET/MRI in lower grade, more favorable prostate cancer presents an opportunity to reduce initial misclassification of disease among potential AS and FT candidates. In our pilot clinical trial, the use of ^18^F‐rhPSMA‐7.3‐PET/MRI detected the presence of GG 3–5 disease in 25% of patients with a Decipher score ≥ 0.45. Furthermore, major changes in management plan occurred in 35% of cases, and overall confidence in the final management strategy was significantly improved due to this scan.

The overexpression of PSMA on the extracellular membrane of most prostate cancer cells makes this a remarkable target for diagnostic imaging. Small, radioactively labeled molecules which recognize PSMA, including ^68^Ga‐PSMA‐11, ^18^F‐DCFPyL, and ^18^F‐rhPSMA‐7.3, have gained approval by the Food and Drug Administration (FDA) for the detection of metastatic disease on both initial baseline evaluation and biochemical recurrence. Incidentally, PSMA‐PET/CT was shown to detect 90% of intraprostatic lesions during baseline staging [16]. However, it remained unclear whether these PET‐avid intraprostatic lesions provided any extra information over conventional mpMRI findings. In this context, Australian investigators examined whether pelvic ^68^Ga‐PSMA‐PET/CT provided additional diagnostic value to prostate mpMRI in a cohort of MRI‐naïve patients [17]. They found that a combined PSMA‐PET/CT and mpMRI approach significantly reduced the number of false negative results when compared to mpMRI alone. These findings prompted our interest in utilizing PSMA‐based tracers along with MRI to better understand the intraprostatic disease burden of men who might be candidates for AS or FT.

In this study, we selected ^18^F‐rhPSMA‐7.3 for PSMA‐PET imaging. Though head‐to‐head studies between PSMA‐PET tracers are limited, detection rates of distant prostate cancer with ^18^F‐rhPSMA‐7.3‐PET/CT compare similarly to other tracers but with significantly lower than average urinary excretion [18, 19, 20, 21]. This decreases the intensity of uptake within the urinary bladder and potentially decreases the volume averaging artifact on the prostate gland, seminal vesicles, and adjacent lymph nodes. The improved discrimination between cancerous lesions and urinary excretion bolstered our case for selecting ^18^F‐rhPSMA‐7.3 as an intraprostatic disease marker. Furthermore, this radiotracer has been associated with very high inter‐reader reproducibility for intraprostatic lesions among patients undergoing imaging in the primary staging setting [22]. Our results demonstrated an extreme reclassification rate of 25% among potential AS candidates. While similar results may be achieved using other PSMA imaging agents, that has yet to be determined. It is important to note that the cohort for our pilot study was intentionally selected for AS candidates with more aggressive disease biology (i.e., Decipher ≥ 0.45) to determine at least evidence of possible utility of PSMA‐PET imaging in this patient population. This Decipher threshold was used to enrich the study with patients who may stand to benefit the most from additional testing. Expanded studies will be necessary to determine if PET imaging has clinical utility in all men considering AS or FT, regardless of disease biology, and to determine the value of PET imaging in men who were not preselected, as the financial burden of PET imaging can be high.

Concerns about mischaracterizing disease burden and risk fuel the anxiety for both clinicians and patients. Even among cases in which upgrading or upstaging was absent, we found that confidence in the final treatment plan was significantly improved after performing the PSMA‐PET/MRI. Indeed, the presence of a Decipher score ≥ 0.45 for a patient considering AS or FT may introduce doubts due to the suggestion of more aggressive disease biology. Given that 10%–20% of patients on AS undergo treatment without evidence of disease reclassification, these advanced confirmatory tests may provide the reassurance needed to combat early dropout [23, 24]. Treatment planning for FT represents another space in which PSMA‐PET/MRI may show benefit. Perhaps even more than AS, the success of FT hinges on accurate detection and localization of disease. To this end, the Focal Therapy Consensus (FALCON) group has published best practice statements which necessitate the use of mpMRI prior to FT [25]. However, clinically significant lesions can be missed on MRI in 10%–30% of cases [26, 27, 28]. This missed detection likely contributes, at least in part, to the 2‐year recurrence rate of about 20% after FT, with out‐of‐field recurrences comprising slightly over half of cases [29]. In the present study, we found that the use of PSMA‐PET/MRI increased confidence in treatment strategy for the 5 patients who elected to undergo FT.

Our study has several limitations. As a pilot clinical trial, our cohort was limited to 20 patients to evaluate for clinical significance. Accordingly, these patients were selected to be higher risk AS candidates by virtue of their intermediate to high risk Decipher scores, as well as the majority of them harboring favorable intermediate risk disease at enrollment. Furthermore, it cannot be definitively determined whether upgrading was seen due to progression of the disease process or due to initial misclassification of disease. However, with a median time of 5 months between pre‐enrollment biopsy and post‐imaging pathology, it is unlikely that the disease progressed in any meaningful manner. Finally, patients who did not undergo radical prostatectomy may continue to harbor occult adverse pathology that was not detected on PSMA‐PET/MRI‐guided biopsy. However, as a pragmatic trial in patients who are candidates for AS or FT, radical prostatectomy for all patients would have been an unrealistic mandate.

## 5 Conclusions

In this pilot clinical trial, ^18^F‐rhPSMA‐7.3‐PET/MRI was shown to be safe and effective at detecting occult higher risk disease in a cohort of potential AS and FT candidates. The information gained from the PSMA‐PET/MRI prompted major changes in management in one‐third of cases, with the majority opting for treatment. Furthermore, PSMA‐PET/MRI significantly increased confidence in treatment plans, which may have implications in reducing AS‐associated anxiety, selecting men for FT, and reducing treatment‐related regret. Given our findings, expanded investigations into PSMA‐PET‐based imaging in favorable risk men with localized prostate cancer are warranted.

## Conflicts of Interest

Clinical trial support was provided by Blue Earth Diagnostics Inc. Ashley E. Ross is a consultant for Blue Earth Diagnostics Inc. No other authors declare a conflict of interest.

## Data Availability

The data that support the findings of this study are available from the corresponding author upon reasonable request.

## References

[1] E. M. Schaeffer , S. Srinivas , N. Adra , et al., “Prostate Cancer, Version 3.2024: Featured Updates to the NCCN Guidelines,” Journal of the National Comprehensive Cancer Network 22, no. 3 (2024): 140–150, 10.6004/jnccn.2024.0019. 38626801

[2] J. A. Eastham , G. B. Auffenberg , D. A. Barocas , et al., “Clinically Localized Prostate Cancer: AUA/ASTRO Guideline, Part I: Introduction, Risk Assessment, Staging, and Risk‐Based Management,” Journal of Urology 208, no. 1 (2022): 10–18, 10.1097/JU.0000000000002757. 35536144

[3] C. H. Walker , K. A. Marchetti , U. Singhal , and T. M. Morgan , “Active Surveillance for Prostate Cancer: Selection Criteria, Guidelines, and Outcomes,” World Journal of Urology 40, no. 1 (2022): 35–42, 10.1007/s00345-021-03622-8. 33655428

[4] L. F. Newcomb , J. M. Schenk , Y. Zheng , et al., “Long‐Term Outcomes in Patients Using Protocol‐Directed Active Surveillance for Prostate Cancer,” Journal of the American Medical Association 331, no. 24 (2024): 2084–2093, 10.1001/jama.2024.6695. 38814624 PMC11140579

[5] M. A. Diven , L. Tshering , X. Ma , et al., “Trends in Active Surveillance for Men With Intermediate‐Risk Prostate Cancer,” JAMA Network Open 7, no. 8 (2024): e2429760, 10.1001/jamanetworkopen.2024.29760. 39172448 PMC11342134

[6] R. Alam , H. B. Carter , P. Landis , J. I. Epstein , and M. Mamawala , “Conditional Probability of Reclassification in an Active Surveillance Program for Prostate Cancer,” Journal of Urology 193, no. 6 (2015): 1950–1955, 10.1016/j.juro.2014.12.091. 25572035 PMC4696016

[7] J. von Hardenberg , A. Borkowetz , F. Siegel , et al., “Potential Candidates for Focal Therapy in Prostate Cancer in the Era of Magnetic Resonance Imaging‐Targeted Biopsy: A Large Multicenter Cohort Study,” European Urology Focus 7, no. 5 (2021): 1002–1010, 10.1016/j.euf.2020.09.015. 33877047

[8] K. J. Tay , “Focal Therapy for Prostate Cancer‐Ready to Be a Standard of Care?,” Prostate Cancer and Prostatic Diseases 24, no. 4 (2021): 931–932, 10.1038/s41391-021-00376-7. 34007016

[9] A. Herlemann , H. C. Huang , R. Alam , et al., “Decipher Identifies Men With Otherwise Clinically Favorable‐Intermediate Risk Disease Who May Not Be Good Candidates for Active Surveillance,” Prostate Cancer and Prostatic Diseases 23, no. 1 (2020): 136–143, 10.1038/s41391-019-0167-9. 31455846 PMC8076042

[10] H. L. Kim , P. Li , H. C. Huang , et al., “Validation of the Decipher Test for Predicting Adverse Pathology in Candidates for Prostate Cancer Active Surveillance,” Prostate Cancer and Prostatic Diseases 22, no. 3 (2019): 399–405, 10.1038/s41391-018-0101-6. 30542054 PMC6760567

[11] A. Zhu , J. A. Proudfoot , E. Davicioni , et al., “Use of Decipher Prostate Biopsy Test in Patients With Favorable‐Risk Disease Undergoing Conservative Management or Radical Prostatectomy in the Surveillance, Epidemiology, and End Results Registry,” European Urology Oncology Published Online July 6 S2588–9311, no. 24 (2024): 00154–00158, 10.1016/j.euo.2024.06.007. 38972832

[12] R. Alam , H. B. Carter , J. I. Epstein , and J. J. Tosoian , “Active Surveillance of Prostate Cancer: Current State of Practice and Utility of Multiparametric Magnetic Resonance Imaging,” Reviews in Urology 19, no. 2 (2017): 77–88, 10.3909/riu0757a. 28959144 PMC5610357

[13] M. D. Ho , A. E. Ross , and S. E. Eggener , “Risk Stratification of Low‐Risk Prostate Cancer: Individualizing Care in the Era of Active Surveillance,” Journal of Urology 210, no. 1 (2023): 38–45, 10.1097/JU.0000000000003454. 37042807

[14] L. Lindenberg , M. Ahlman , B. Turkbey , E. Mena , and P. Choyke , “Evaluation of Prostate Cancer With PET/MRI,” Journal of Nuclear Medicine 57, no. Suppl 3 (2016): 111S–116S, 10.2967/jnumed.115.169763. 27694163 PMC12530357

[15] I. Berger , C. Annabattula , J. Lewis , et al., “68Ga‐PSMA PET/CT vs. mpMRI for Locoregional Prostate Cancer Staging: Correlation With Final Histopathology,” Prostate Cancer and Prostatic Diseases 21, no. 2 (2018): 204–211, 10.1038/s41391-018-0048-7. 29858591

[16] M. Perera , N. Papa , M. Roberts , et al., “Gallium‐68 Prostate‐Specific Membrane Antigen Positron Emission Tomography in Advanced Prostate Cancer‐Updated Diagnostic Utility, Sensitivity, Specificity, and Distribution of Prostate‐Specific Membrane Antigen‐Avid Lesions: A Systematic Review and Meta‐Analysis,” European Urology 77, no. 4 (2020): 403–417, 10.1016/j.eururo.2019.01.049. 30773328

[17] L. Emmett , J. Buteau , N. Papa , et al., “The Additive Diagnostic Value of Prostate‐Specific Membrane Antigen Positron Emission Tomography Computed Tomography to Multiparametric Magnetic Resonance Imaging Triage in the Diagnosis of Prostate Cancer (PRIMARY): A Prospective Multicentre Study,” European Urology 80, no. 6 (2021): 682–689, 10.1016/j.eururo.2021.08.002. 34465492

[18] T. Tolvanen , K. Kalliokoski , S. Malaspina , et al., “Safety, Biodistribution, and Radiation Dosimetry of 18F‐rhPSMA‐7.3 in Healthy Adult Volunteers,” Journal of Nuclear Medicine 62, no. 5 (2021): 679–684, 10.2967/jnumed.120.252114. 33067338 PMC8844263

[19] A. B. Jani , G. C. Ravizzini , B. A. Gartrell , et al., “Diagnostic Performance and Safety of 18F‐rhPSMA‐7.3 Positron Emission Tomography in Men With Suspected Prostate Cancer Recurrence: Results From a Phase 3, Prospective, Multicenter Study (SPOTLIGHT),” Journal of Urology 210, no. 2 (2023): 299–311, 10.1097/JU.0000000000003493. PMC12721651 37126069

[20] D. S. Surasi , M. Eiber , T. Maurer , et al., “Diagnostic Performance and Safety of Positron Emission Tomography With 18F‐rhPSMA‐7.3 in Patients With Newly Diagnosed Unfavourable Intermediate‐ to Very‐High‐Risk Prostate Cancer: Results From a Phase 3, Prospective, Multicentre Study (LIGHTHOUSE),” European Urology 84, no. 4 (2023): 361–370, 10.1016/j.eururo.2023.06.018. 37414702

[21] P. H. Kuo , R. Hermsen , R. Penny , and E. J. Postema , “Quantitative and Qualitative Assessment of Urinary Activity of 18F‐Flotufolastat‐PET/CT in Patients With Prostate Cancer: A Post Hoc Analysis of the LIGHTHOUSE and SPOTLIGHT Studies,” Molecular Imaging and Biology 26, no. 1 (2024): 53–60, 10.1007/s11307-023-01867-w. 37932609 PMC10827967

[22] P. H. Kuo , G. Esposito , G. A. Ulaner , et al., “Interreader and Intrareader Reproducibility of 18F‐Flotufolastat Image Interpretation in Patients With Newly Diagnosed or Recurrent Prostate Cancer: Data From Two Phase 3 Prospective Multicenter Studies,” Journal of Nuclear Medicine 65, no. 8 (2024): 1239–1243, 10.2967/jnumed.123.267306. 38871390 PMC11294070

[23] P. S. Kirk , K. Zhu , Y. Zheng , et al., “Treatment in the Absence of Disease Reclassification Among Men on Active Surveillance for Prostate Cancer,” Cancer 128, no. 2 (2022): 269–274, 10.1002/cncr.33911. 34516660 PMC8738121

[24] L. P. Bokhorst , R. Valdagni , A. Rannikko , et al., “A Decade of Active Surveillance in the PRIAS Study: An Update and Evaluation of the Criteria Used to Recommend a Switch to Active Treatment,” European Urology 70, no. 6 (2016): 954–960, 10.1016/j.eururo.2016.06.007. 27329565

[25] L. Rodríguez‐Sánchez , R. Reiter , A. Rodríguez , et al., “The FocAL Therapy CONsensus (FALCON): Enhancing Partial Gland Ablation for Localised Prostate Cancer,” BJU International 134, no. 1 (2024): 50–53, 10.1111/bju.16360. 38613454

[26] S. Borofsky , A. K. George , S. Gaur , et al., “What Are We Missing? False‐Negative Cancers at Multiparametric MR Imaging of the Prostate,” Radiology 286, no. 1 (2018): 186–195, 10.1148/radiol.2017152877. 29053402 PMC5749595

[27] A. Mohammadian Bajgiran , S. Afshari Mirak , S. Shakeri , et al., “Characteristics of Missed Prostate Cancer Lesions on 3T Multiparametric‐MRI in 518 Patients: Based on PI‐RADSv2 and Using Whole‐Mount Histopathology Reference,” Abdominal Radiology 44, no. 3 (2019): 1052–1061, 10.1007/s00261-018-1823-6. 30460528

[28] J. W. Jang , A. Abrams , A. Jawahar , et al., “Detection of MRI‐Invisible Disease Using PSMA PET/CT in a Patient Considering Focal Therapy,” Case Reports in Urology 2025 (2025): 2981515, 10.1155/criu/2981515. 40177325 PMC11964713

[29] K. J. Tay , K. Y. Fong , A. Stabile , et al., “Established Focal Therapy‐HIFU, IRE, or Cryotherapy‐Where Are We Now?—A Systematic Review and Meta‐Analysis,” Prostate Cancer and Prostatic Diseases 28 (2024): 693–706, 10.1038/s41391-024-00911-2. 39468217 PMC12399424

